# Ectopic thymoma of the lung; a rare case report and review of the literature

**DOI:** 10.1186/1757-1626-2-9149

**Published:** 2009-12-04

**Authors:** Kostas Skoutelis, Dimitrios D Nikolopoulos, Kostas Markopoulos, Katerina Chabipi, Sofia Stoungioti, Miltiadis Papastamatiou

**Affiliations:** 1 7th Hospital of Social Security Institute, Surgical Department of Thorax and Vascular Surgery, Kautazoglou 11 str, Athens,111-44, Greece; 2 Central Clinic of Athens, Surgical Department of Thorax and Vascular Surgery, Asklepiou 31 str, Athens, 106-80, Greece; 3 1st Hospital of Social Security Institute, Head Neurologist, Zaimi str, Athens, 151-27, Greece; 4 Central Clinic of Athens, Ophthalmologist, Asklepiou 31 str, Athens, 106-80, Greece; 5 7th Hospital of Social Security Institute, Head Surgeon of 2nd Surgical Department, Kautazoglou 11 str, Athens, 111-44, Greece

## Abstract

**Introduction:**

Ectopic thymoma is a rare neoplasm, which can be developed in various sites, with the lung being amongst the rarest.

**Case presentation:**

In this paper, we present the case of a woman with a slow-growing ectopic thymoma, stemming from the visceral pleura of the upper lobe of the left lung anteriorly, extending into the left lung and the cardiac wall, invading the fatty tissue near the pericardium, notably without infiltrating the lung or cardiac parenchyma. The thymoma was successfully removed via thoracotomy.

**Conclusion:**

Ectopic thymoma is an uncommon neoplasm. To our knowledge, a case of an ectopic thymoma stemming from the visceral pleura of the lung is extremely rare.

## Introduction

Although the vast majority of thymomas are located in the anterior mediastinum (90%), there have been many sites of ectopic localization of thymomas described. Ectopic thymomas have been found in the middle and the posterior mediastinum, the neck, the base of the skull, the pericardium, the lung parenchyma, and the pleural cavity [1-5]; whereas in the visceral pleura are extremely uncommon [6]. In this report, we present a rare case of an asymptomatic female with an ectopic thymoma stemming from the visceral pleura of the left lung.

## Case presentation

**Figure 1:**
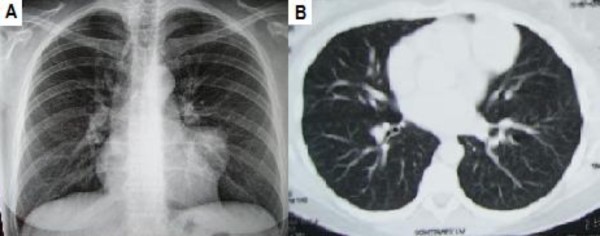
**(a) Pre-operative chest radiography**. (b) Pre-operative CT scan.

In April 2007, a 40-year-old Caucasian woman admitted into our clinic for performing an orthopaedic operation. The chest x-ray, which is amongst the routine pro-operating exams preceding a surgery, revealed an asymptomatic left paramediastinal mass in the left hemithorax (Figure 1a). After a chest CT scan, there was revealed a large, 5.5 cm in diameter, multilobar mass attached to the upper lobe of the left lung, extending into the left lung, touching the cardiac wall and invading the pericardial fatty tissue, without infiltrating the parenchyma of neither the lung nor the heart (Figure 1b). The CT imaging of the abdomen showed no signs of lymph node enlargement or any metastatic disease. Subsequently, a biopsy of the tumour was conducted, through an FNA CT-scan. The histological results revealed a hyperplasia of the lymphoid tissue.

**Figure 2:**
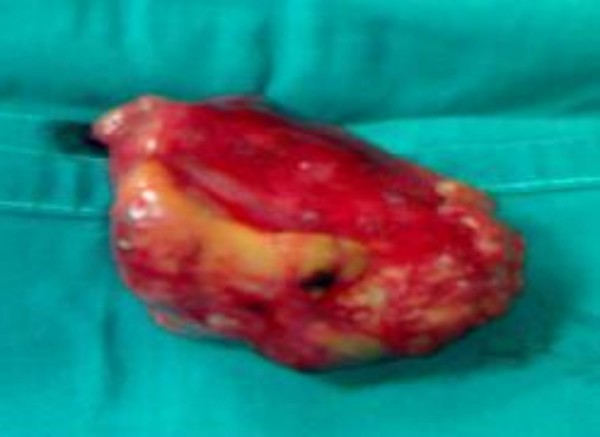
**Thymoma after removed from the anterior periviscelar pleura of the left lung**.

Following these, the patient was scheduled for surgery. She was operated on through a posterolateral thoracotomy in the 5^th ^left intercostal space. A 5 × 6 × 4.5 cm tumour was found with a pedicle stemming from the visceral pleura of the upper lobe of the left lung anteriorly. It was "touching" the wall of the left lung and heart, obscuring the pericardia fatty tissue. There were no signs of the tumour infiltrating the lung or any of the adjacent structures. Note that the only connection of the tumor with the lung was its pedicle and that there was no connection of the tumour with the mediastinum and the thymus. Consequently, the pedicle was sutureligated, divided and removed (Figure 2).

**Figure 3:**
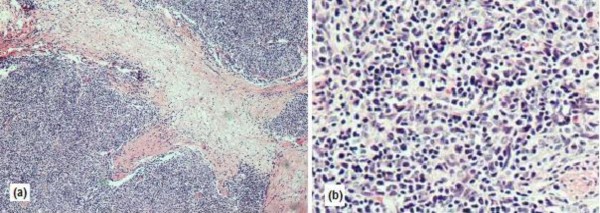
**(a) A solid nest of epithelial cells surrounded by dense lymphoplasmacytic infiltrates**. (b) Microscopic findings of spindle neoplastic epithelial cells of an ectopic thymoma.

The postoperative course was uneventful and the patient was released from the hospital on the 5th post-operative day. The anatomopathological examination of the sample using optical microscopy and immunohistochemical tests confirmed the diagnosis of an ectopic thymoma (Figure 3a, 3b). The resected specimen was a solid and elastic multi-lobulated, grey mass, with a thick fibrous capsule. The microscopy showed nodules of varying sizes comprising a mixture of polygonal, ovoid and spindle (epithelial) cells, associated with a dense lymphoplasmacytic infiltrate, vascular proliferation, nuclear pleomorphism, scanty mitoses, but no tumour giant or necrotic cells. These histopathologic findings were consistent with a type AB according to World Health Organization Classification System (1999) and the pathologic stage was stage I on the classification system of Masaoka (Table 1) [1,7,8].

**Table 1:** Masaoka staging system

**Masaoka staging system **[7]	
**STAGE I**	Macroscopically encapsulated with no microscopically detectable capsular invasion.
**STAGE II**	Macroscopic invasion of mediastinal fatty tissue or mediastinal pleura or microscopic invasion into the capsule.
**STAGE III**	Macroscopic invasion into surrounding structures (pericardium, great vessels, lung).
**STAGE IV**	(A) Pleural or pericardial dissemination.
	(B) Lymphogenous or hematogenous metastases.

## Discussion

The thymus gland is an anterior mediastinal structure arising embryologically from the third, and to a lesser extent, the fourth pharyngeal pouch. A prominent organ early in life, the thymus reaches a maximum size of 40 g during puberty before regressing and involuting during adulthood, being replaced by fibro-fatty tissue [1]. Thymoma is an uncommon neoplasm that derives from the epithelial cells of thymus and it is the most common neoplasm of the anterior mediastinum, most frequently found in adults at their 40 s. Nevertheless, cases of *ectopic thymomas *have been found in various locations, as it has already mentioned [1-6].

Furthermore, the thymomas have many interesting clinical features, such as their unique association with myasthenia gravis or autoimmune diseases (as pancytopenia, hypogammaglobulinemia). Approximately 35% of thymoma patients have myasthenia gravis, while 5%-10% have other systemic syndromes [1,8]. Moreover, in the case of an ectopic thymoma, the possibility of solitary metastases from a small undetected primary tumour in the thymus should be considered [8,9].

The majority of the patients suffering from thymoma are asymptomatic. However, when clinical symptoms are present, they can roughly be differentiated between *localizing symptoms*, and *systemic symptoms*, due to the release of excess hormones, antibodies, and cytokines [1,8,9]. The most common clinical symptoms are cough (60%), chest pain (30%), fevers/chills (20%) and dyspnea (16%) [1,8,9]. Myasthenia gravis patients are characterized by the development of autoimmune antibodies to the acetylcholine receptor on postsynaptic neuromuscular junctions. These antibodies could be detected with blood tests [8].

Nevertheless, imaging is an essential part of the workup and in conjunction with history and physical exam is often the only investigation needed prior to treatment [1]. Following identification of a mass on chest x-ray, a CT scan of the chest should be obtained, as it allows for the characterization of tumors as well as an assessment of possible invasion into surrounding structures [10]. Dynamic magnetic resonance imaging (MRI) has been examined as a potential way to improve staging and differential diagnosis determination [11], and PET has been examined for tumor detection and differentiation between invasive and noninvasive thymoma with mixed results [12].

The thymomas can be classified according to the degree of invasion of the tumor through the capsule into the surrounding structures (*Masaoka staging system *- the most widely accepted) or according to the morphology of the epithelial cells and the lymphocyte-to-epithelial cell ratio (WHO system) [7-9]. Surgical resection is the standard of care for both non-invasive and invasive thymomas as it provides the best prognosis [13]. For locally invasive or metastatic disease, or inoperable tumors, adjunctive therapy is used, which may include chemotherapy and radiation treatment [1,8,9,13].

Completely resected, Masaoka stage II and III thymomas may benefit from adjuvant radiotherapy to reduce local recurrence rates, but without impact on survival. In primary unresectable thymomas, multimodal strategy nowadays includes neo-adjuvant chemotherapy, extensive surgery, adjuvant radiotherapy, and in some cases, adjuvant chemotherapy. The most popular chemotherapy regimens combine cisplatin, adriamycin, etoposide, cyclophophamide, or ifosfamide [14,15].

Due to thymomas rarity, the most chemotherapeutic trials are case reports or phase II trials, while prospective randomized trials are not available, so as to compare the different chemotherapeutic agents. Nevertheless, the most commonly employed drug in combination chemotherapy of thymomas is cisplatin and several studies reported responses in excess of 50% with these combinations [8,13-15].

## Conclusion

Ectopic thymoma is an uncommon neoplasm. To our knowledge, a case of an ectopic thymoma stemming from the visceral pleura of the lung is extremely rare.

## Consent

Written informed consent was obtained from the patient for publication of this case report and accompanying images. A copy of the written consent is available for review by the Editor-in-Chief of this journal.

## Competing interests

The authors declare that they have no competing interests.

## Authors' contributions

KS conducted a literature search, prepared the final manuscript and operated the patient. DDN conducted a literature search, contributed to the preparation of the manuscript and operated the patient. MK prepared the first draft of the manuscript and treated the patient. CK, SS and MP supervised the manuscript, examined and treated the patient. KS, DDN and KM participated in the surgical operation, and contributed equally in the post-operative patient's follow-up. All authors contributed in collecting patient data and editing arthroscopic operative images. All authors read and approved the final manuscript.
